# 

**Published:** 1914-11-28

**Authors:** 


### A "Unique Record" as Senior Surgeon.

SIXTY YEARS AT SUNDERLAND INFIRMARY.

On the retirement of Dr. G. B. Morgan, J.P., senior surgeon at the Royal Infirmary, Sunderland, the house committee addressed to him a letter, from which we take the following extracts: "Among the many friends who will be wishing you well on the attainment of your eightieth birthday the members of the house committee at the Royal Infirmary desire to find a place. To have far out-paced the allotted span, with surprising vigour of body and mind, is an achievement which must awaken a reverent gratitude in any man, even as it constrains congratulation from all his comrades. We realise also that you have chosen so signal a season to bring to a close your long and honourable connection with the Royal Infirmary—a relationship as fruitful as it has been faithful. To have spent well-nigh sixty years in unbroken and devoted service to this great house of suffering, manifesting a kind and practical interest in every department of its varied work, is, we believe, a record unique in the history of our institution, if, indeed, it can be paralleled by that of any other."

### A Hospital Manager as Mayor.

Alderman Jonathan North, J.P., who has been elected Mayor of the Borough of Leicester, becomes *ex-officio* a member of the Board of Governors of the Leicester Royal Infirmary. The new Mayor has for some years been closely associated with Sir Edward Wood (the chairman of the infirmary) in the great Leicester business of Freeman, Hardy and Willis, Ltd., whose benefactions to local philanthropic institutions have been marked by great generosity. Alderman North has for many years played a prominent part in the municipal life of the borough. For the past eight years he has occupied the chair of the Education Committee. During that time the work of the committee has been marked by great developments, and under Alderman North's guidance considerable impetus has been given to the progress of secondary education and evening continuation schools in the borough.

### Buildings Contemplated.

AYLESBURY.—The Aylesbury Urban District Council invite tenders for the erection of a ward and bathroom at the isolation hospital. Quantities may be obtained of Mr. W. H. Taylor, engineer, Town Hall, Aylesbury.

BARNES.—The Barnes Urban District Council invite tenders for the erection of a dispensary and tuberculosis wards at the hospital. Quantities may be obtained of Mr. G. B. Tones, surveyor, Council House, High Street, Mortlake.

BRACEBRIDGE.—The Lindsey County Council have approved plans for extensions of the Bracebridge Asylum at a cost not to exceed £40,000. It is proposed to construct two wings, with airing courts, boundary walls, roads, etc.

CHORLEY.—The Guardians have adopted amended plans prepared by Mr. Harrison for new vagrant wards, including a self-contained heating installation. The estimated cost is £4,377.

DUBLIN.—At the last meeting of the Incorporated Dental Hospital Board at 22 Lincoln Place, it was stated that a contract had been entered into with Messrs. Martin to build the new wing of the hospital. The plans are by Messrs. Orpen and Dickenson, and the cost will be £2,800.

LEEDS.—The Committee of the Leeds General Infirmary invite tenders for the erection of new operating theatres block, new ward pavilion block, and extensions to the nurses' home. Mr. Sidney D. Kitson, Lloyds Bank Chambers, Vicar Lane, Leeds, is the architect, from whom quantities may be obtained.

**Rates of Subscription**: Twelve months, 6/6; Six months, 4/-; Three months, 2/-, post free to any address in the United Kingdom. Abroad, Twelve months, 8/8; Six months, 5/-; Three months, 2/6, post free.—Address The Subscription Dept., "The Hospital," The Hospital Building, 28/29 Southampton Street, Strand, London W.C.

